# Buddha’s Tooth

**Published:** 1899-09

**Authors:** 


### Buddha's Tooth.

There have been many Buddhist ceremonies in Colombo and recently Kandy in connection with the landing of the golden casket, presented by the Buddhists of Burma for enclosing the famous tooth of Buddha, whose resting place is the great Mahigawa Temple at Kandy. The value of this magnificent casket is a lakh and a half of rupees (£10,000). It is a wonderful piece of workmanship in the shape of a dagoba. The body is of massive gold and is garlanded with strings of jewels and surmounted by a splendid ruby. It is covered by a silver canopy inlaid with precious stones, and the whole stands about six feet high. With it came from Rangoon thirteen hundred Burmese, of whom three hundred and seventy were priests. An interesting member of the party was an old lady worth £250,000 in worldly goods, who had herself contributed over £6,000 toward the gift. It was kept in her cabin during the voyage and it is said she sat upon it all the way! The Archbishop and several Burmese princesses were also of the party. The ardor of the local Buddhists was somewhat dampened when they found they had to pay £280 duty on their new treasure. The cask has not yet been moved to Kandy where it will go in the great state, but that place is frightfully overcrowded with pilgrims awaiting its arrival, all eager to view the tooth relic—so much so that the military have been called out to preserve order in the precincts of the temple.

